# Health state utility values for diabetic retinopathy: protocol for a systematic review and meta-analysis

**DOI:** 10.1186/s13643-015-0006-6

**Published:** 2015-02-21

**Authors:** Christopher J Sampson, Jonathan C Tosh, Christopher P Cheyne, Deborah Broadbent, Marilyn James

**Affiliations:** Division of Rehabilitation and Ageing, School of Medicine, University of Nottingham, Queen’s Medical Centre, Nottingham, NG7 2UH UK; School of Health and Related Research, University of Sheffield, Regent Court, Sheffield, S1 4DA UK; Department of Biostatistics, University of Liverpool, Daulby Street, Liverpool, L69 3GA UK; Department of Eye and Vision Science, Royal Liverpool University Hospital, Prescot Street, Liverpool, L7 8XP UK

**Keywords:** Systematic review, Utility, Economic evaluation, Cost-utility analysis, Diabetic retinopathy, Meta-regression, Modeling, Health technology assessment

## Abstract

**Background:**

People with diabetic retinopathy tend to have lower levels of health-related quality of life than individuals with no retinopathy. Strategies for screening and treatment have been shown to be cost-effective. In order to reduce the bias in cost-effectiveness estimates, systematic reviews of health state utility values (HSUVs) are crucial for health technology assessment and the development of decision analytic models. A review and synthesis of HSUVs for the different stages of disease progression in diabetic retinopathy has not previously been conducted.

**Methods/Design:**

We will conduct a systematic review of the available literature that reports HSUVs for people with diabetic retinopathy, in correspondence with current stage of disease progression and/or visual acuity. We will search Medline, EMBASE, Web of Science, Cost-Effectiveness Analysis Registry, Centre for Reviews and Dissemination Database, and EconLit to identify relevant English-language articles. Data will subsequently be synthesized using linear mixed effects modeling meta-regression. Additionally, reported disease severity classifications will be mapped to a four-level grading scale for diabetic retinopathy.

**Discussion:**

The systematic review and meta-analysis will provide important evidence for future model-based economic evaluations of technologies for diabetic retinopathy. The meta-regression will enable the estimation of utility values at different disease stages for patients with particular characteristics and will also highlight where the design of the study and HSUV instrument have influenced the reported utility values. We believe this protocol to be the first of its kind to be published.

**Systematic review registration:**

PROSPERO CRD42014012891

**Electronic supplementary material:**

The online version of this article (doi:10.1186/s13643-015-0006-6) contains supplementary material, which is available to authorized users.

## Background

Diabetic retinopathy is one of the most common causes of blindness [1,2], but effective and cost-effective preventive and remedial interventions are available [3,4]. When a new technology is developed, an economic evaluation is often conducted to determine whether it is cost-effective. It is common for such economic evaluations to require decision modeling, where evidence is synthesized to determine long-term costs and health benefits. In order to minimize bias in these decision problems, it is crucial that the process of selecting the evidence for the model is robust, transparent, and systematic [5]. This is a requirement of the National Institute for Health and Care Excellence (NICE), which publishes guidance for the NHS based on the clinical and cost-effectiveness evidence for health technologies [6]. NICE identify quality-adjusted life years (QALYs) as their preferred measure of health outcome; an economic evaluation using QALYs is known as a cost-utility analysis. QALYs combine quantity and quality of life, and the quality aspect is captured using health state utility values (HSUVs). HSUVs are generally measured on a scale anchored at 1 and 0, where 1 corresponds to ‘full health’ and 0 represents a health state of equivalent value to being dead.

It has been identified that many submissions to NICE do not satisfy their requirements with regard to the transparent and systematic selection of HSUVs [7]. A growing number of systematic reviews are being carried out to inform better selection of HSUVs [8-28], with some synthesizing the data using meta-analysis [9-12,14,15,17,18,22,24-26]. Such an approach is increasingly being seen as an important step in the process of a model-based economic evaluation [29].

In modeling studies of diabetic retinopathy, some researchers choose HSUVs based on estimated visual acuity levels, rather than on the disease state itself [28,30]. It is unclear whether or not the effect of visual acuity on HSUVs is consistent across different visual disorders [31]. Any given level of visual acuity may be associated with different levels of health-related quality of life because of the diverse impacts of disease on vision; whether the effect is on central or peripheral vision may be of greater import, for example. Therefore, such an approach is unlikely to be valid in accurately estimating the impact of an intervention on health-related quality of life. For diabetic retinopathy, treatment pathways are usually defined by severity of disease, rather than visual acuity. As such, most modeling studies differentiate between health states based on this and therefore need appropriate HSUVs for these disease states.

Disease progression in diabetic retinopathy (DR) is classified in a number of ways. Often these classifications relate to whether the disease is proliferative (PDR) or non-proliferative (NPDR). In the USA, the most common classification system has five or six levels (no DR/mild NPDR/moderate NPDR/severe NPDR/PDR/PDR with high-risk characteristics), as proposed by the American Academy of Ophthalmology [32]. The NHS Diabetic Eye Screening Programme (NDESP) in the UK uses four levels (no DR/background DR/pre-proliferative DR/PDR) [33]. The Early Treatment Diabetic Retinopathy Study used a more detailed grading system [34]. It is also possible to classify disease based on outcome or management pathways; the Liverpool Diabetic Eye Study, for example, uses seven levels relating to screening, referral and treatment [35]. Other systems include the Scottish Diabetic Retinopathy Grading Scheme, the Royal College of Ophthalmologists grading system and feature-specific grading.

We will carry out a systematic review and meta-analysis in order to identify whether or not individuals with different levels of diabetic retinopathy have different HSUVs and, if they do, what these levels are. Although our focus is upon HSUVs associated with specific stages of disease progression in DR, we will also review HSUVs associated with visual function in people with DR. A meta-analysis will be carried out to enable modelers to estimate the most appropriate HSUVs with which to populate their models. The primary purpose of this review will be to inform the development of a decision model to evaluate alternative strategies in screening for diabetic retinopathy in a general population of people with diabetes. However, the study is designed such that it can inform future model-based evaluations of any intervention for diabetic retinopathy. The study as such has a number of aims:

1. To provide a narrative overview of published studies reporting HSUVs for diabetic retinopathy.
2. To derive pooled estimates for HSUVs that correspond to disease states based on the most commonly used disease classification systems.
3. To quantify the effects on reported HSUVs of variation in study design.
4. To map reported values to a consistent grading scale for use in a modeling study.

## Methods/Design

Guidelines such as the PRISMA statement [36] are not wholly applicable to the review, though we have developed our methods in line with published recommendations [37-41] where appropriate. The Patient, Intervention, Comparison, Outcome (PICO) question is not usually applicable to reviews of health state utility values [29]. For example, this review should not focus on a specific intervention or comparison. Furthermore, it is also necessary to define additional requirements; for example, it is important to define which HSUV elicitation methods will be included. This protocol is registered with Prospective Register of Systematic Reviews (PROSPERO; registration number: CRD42014012891). Our analysis will not require approval from an ethics committee or written informed consent because it relies entirely on published sample-level data.

### Search strategy

Our search will be necessarily broad due to inconsistency in the nature and reporting of studies that include HSUVs. Databases for searching will include Medline, EMBASE, Web of Science, Cost-Effectiveness Analysis Registry, Centre for Reviews and Dissemination Database, and EconLit. Specific pre-defined thesaurus terms for HSUVs do not exist, though broader terms may apply. Our search will use general, instrument-specific and method-specific terms, which will be combined with terms for diabetic retinopathy. Diabetic retinopathy is a term which is used to describe progressive retinal changes (for example no DR/background DR/preproliferative DR/PDR) but is also used broadly to cover diabetic retinopathy and maculopathy. We will include studies that report HSUVs for maculopathy. Given that HSUVs are often reported as secondary outcomes, it is likely that they will not be mentioned in titles or abstracts. As such it will be necessary to carry out full text searches. Additional file 1 outlines the search terms we will use. No date or language restrictions will be applied to the electronic searches. We will subsequently carry out citation searches and author searches based on the identified papers. Colleagues will be asked to comment on the completeness of the final list of studies. We will additionally carry out a search for grey literature using our specified terms on a number of specific tools: SHERPA Search [42], Research Papers in Economics [43], and Open Grey [44].

### Study eligibility

#### Inclusion criteria

Studies of any design will be included, and it is expected that all will be categorized as either clinical decision analyses or outcomes studies. Clinical decision analysis studies will include randomized controlled trials and economic evaluations, while outcomes studies are those designed specifically to elicit HSUVs.

Studies must use a recognized method of direct (for example, standard gamble or time tradeoff) or indirect (for example, EQ-5D or Health Utilities Index) utility assessment. HSUVs from visual analog scales, mapping algorithms, and expert opinion will also be included. The language of publication must be English.

All types of publication (both full publications and abstracts) will be included. The population will be people with diabetic retinopathy or those attending screening for diabetic retinopathy. There are no inclusion criteria relating to specific interventions or technologies. The comparator element of the PICO statement does not apply. The studies must report either mean or median HSUVs and these must relate either to visual function or disease states specific to diabetic retinopathy. Such disease states are likely to be based on the grading systems described above, though studies adopting other retinopathy grading systems will be included.

#### Exclusion criteria

We will exclude editorials, reviews, and meta-analyses that do not report original data. Studies that report data from health state classification systems but do not estimate HSUVs will be excluded.

### Data collection

#### Study selection

The first reviewer (CJS) will assess studies for retrieval based on titles and abstracts. The second reviewer (JCT) will check the validity of exclusions. Any disagreements will be resolved by discussion. It is likely that many titles and abstracts will not mention HSUVs, despite their inclusion. Indeed, it has been reported that rejecting studies based on title and abstract can result in lost citations when reviewing HSUVs [37]. Articles will be rejected based on the title and abstract only if it is clear that the study could not have recorded the necessary data for inclusion. Full texts will be retrieved for studies not rejected at abstract screening. These will be assessed by the first reviewer for satisfaction of the inclusion and exclusion criteria. Reasons for exclusion will be recorded, and the second reviewer will check the validity of exclusions. The number of records identified, retrieved, screened, assessed, included, and excluded in the review, and reasons for exclusions, will be summarized in a PRISMA flow diagram [36].

#### Data extraction and management

Data will be extracted by the first reviewer (CJS) using the electronic data extraction form shown in Additional file 2, and automatically recorded in a spreadsheet. A separate form will be completed for each reported HSUV as studies may include different subpopulations; for example, from different countries. Extracted data will subsequently be crosschecked by the second reviewer (JCT). Any disagreements will be resolved by discussion until consensus is reached. Based on previous reviews of HSUVs (cited above), we will record the following for each study: first author, publication year, study title, publication name, study design, interventions/comparators, sample size, and the number of separate HSUVs reported in the paper. For each reported HSUV, we will record point estimate type (mean/median), reported HSUV, measure of variance type (standard deviation/variance), reported variance statistic, retinopathy state, maculopathy state, grading system used, visual function measurement method, visual acuity/function level, sample size, country, age range, other sample specifics, valuation method, valuation source, value set country, upper anchor, lower anchor, administration method, study arm, and treatment status. Where data are reported for individuals without retinopathy - for example, for those attending screening - these data will also be recorded. The data extraction form has been successfully piloted on three pre-identified studies. Where necessary data for analysis are missing, we will attempt contact with the corresponding author.

#### Quality and relevance assessment

Standard means of assessing quality in systematic reviews are not appropriate for reviews of HSUVs, as they may be at odds with the quality of the evidence reported. For example, though randomized controlled trial data may be the ‘gold standard’ for capturing treatment effects, such a study design may be inferior when eliciting HSUVs due to low external validity or lack of relevance. There is limited guidance for assessing the quality of studies reporting HSUVs. We use the hierarchy of data sources presented by Cooper and colleagues [45] to rank studies from 1 to 6, as shown in Table 1. In order to enable researchers to judge the quality of the study, our data extraction form will additionally record as follows: study sample size, inclusion/exclusion criteria, response rates, loss to follow-up, missing data, and the Cooper rank.

**Table 1:** **Cooper rank for utilities (reproduced from Cooper et al. [** 45 **])**

**Rank**	**Data components**
1	Direct utility assessment for the specific study from a sample either:
	(a) of the general population
	(b) with knowledge of the disease(s) of interest
	(c) of patients with the disease(s) of interest
	Indirect utility assessment from specific study from patientsample with disease(s) of interest, using a tool validated for thepatient population
2	Indirect utility assessment from a patient sample with disease(s) of interest, using tool not validated for the patient population
3	Direct utility assessment from a previous study from a sample either
	(a) of the general population
	(b) with knowledge of the disease(s) of interest
	(c) of patients with the disease(s) of interest
	Indirect utility assessment from previous study from patientsample with disease(s) of interest, using a tool validated for thepatient population
4	Unsourced utility data from previous study - method of elicitation unknown
5	Patient preference values obtained from a visual analog scale
6	Delphi panels, expert opinion

The relevance of studies to particular research questions may be more important than quality. For example, the extent to which a study’s results can be used to satisfy the NICE reference case may be crucial. Furthermore, the determinants of relevance may differ for future users of the review. Our data extraction form will record information that will enable users of the review to judge the relevance of the reported HSUV, namely information on interventions/comparators, inclusion/exclusion criteria, country, age range, other sample specifics, valuation method, and valuation source. There is currently no accepted generalizable method for assessing the relevance of HSUVs for a particular study. As such, these data will be summarized qualitatively.

### Data synthesis and presentation

All HSUVs and the characteristics of their associated studies will be tabulated. No qualitative synthesis of the data will be carried out. Saramago et al. identify that quantitative synthesis of aggregate preference-based HSUVs is limited by i) between-study heterogeneity in instruments used, ii) the value set used to quantify utilities, and iii) the models used to approximate scores for health states [39]. Furthermore, a previous review, which reviewed HSUVs associated with different visual acuity levels in diabetic retinopathy, found variation in the methods of elicitation [28]. It is important to measure the effect of these methodological differences on HSUV estimates, and meta-regression is the best way of doing this. Our prior knowledge of the literature suggests that our review will provide sufficient data to carry out an analysis of this kind. A meta-regression model will enable us to predict an expected HSUV for any given set of study characteristics.

For the most commonly used retinopathy grading systems, HSUVs will be pooled, with observations weighted by the inverse of the variance of the mean HSUV, such that:

(1) $$ Y_{i}=y_{i}\left(\frac{n_{i}}{{\sigma^{2}_{i}}}\right),  $$

where *y*_*i*_ is the observed average HSUV and *Y*_*i*_ the weighted average when *n*_*i*_ is the number of respondents and ${\sigma ^{2}_{i}}$ the observed variance of *y*_*i*_. In order to address the limitations of HSUV synthesis previously identified [39], we will use linear mixed-effects modeling to account for fixed and random effects associated with between-study heterogeneity. This methodology is consistent with previous studies [9,24]. The model will allow for random variation on three levels: i) variation between mean HSUVs across studies, ii) variation between mean HSUVs across groups of individuals within studies, and iii) error variation. Studies will report multiple HSUVs, so the meta-regression will adopt a hierarchical approach such that:

(2) $$ Y_{ijk}=\beta_{0}+\sum_{h}\beta_{h}x_{hijk}+v_{i}+u_{ij}+\varepsilon_{ijk},  $$

where *Y*_*ijk*_ is the weighted mean of the *k*th HSUV of the *j*th group being estimated for study *i*, *x*_*ijk*_ are the variables used to explain the between study heterogeneity, *v*_*i*_ is the random effects term of study *i*, *u*_*ij*_ is the random effects term for the *j*th group of study *i*, and *ε*_*ijk*_ is the random error term with fixed variance to be estimated.

Predictor variables will be generated to include retinopathy state, maculopathy state, publication year, study design, country, valuation method, valuation source, and administration method. We will explore the inclusion of other covariates and will use a stepwise procedure of model selection in order to reduce the likelihood of errors. We will test for heteroscedasticity associated with the inclusion of particular predictor variables. Covariates will only be included where the existing evidence suggests that an association with the HSUV outcome might exist. We will estimate variance inflation factors to test for collinearity, and any strongly correlated variables will be removed or collapsed if possible. Selection of variables to be included in *x*_*ijk*_ in the final model will be informed by Akaike’s information criterion. The base case will - as far as possible - match the NICE reference case (that is, use EQ-5D values) [6]. If a study does not have sufficient data for inclusion in the model, the data will be assumed missing at random and the study will be dropped from the model. We will only carry out our proposed modeling work if the data retrieved from the review are sufficient.

Additionally, we will attempt to map values to a disease state classification with four levels of retinopathy and two levels of maculopathy. The mapped value for each HSUV will be recorded using the data extraction sheet and the mapping of the states will subsequently be agreed with a clinician (DB). We will use the same regression methods described above to pool values based on these classifications. We will estimate the intraclass correlation coefficient associated with studies classified in this way when no moderators are included, in order to quantify the heterogeneity associated with such an approach. Publication bias should not be of concern in a review of HSUVs, as they are usually used as a secondary outcome and therefore do not influence the likelihood of publication.

## Discussion

It is common for modeling studies to use utility values from a single study deemed to be most relevant. Guidelines state that the choice of utility values should be transparent and systematic. However, systematic reviews are not common practice and this may result in biased estimates of cost-effectiveness. By reporting all available HSUVs alongside study characteristics, modelers will be able to select the most appropriate value. Furthermore, the results of the meta-regression will enable the estimation of HSUVs based on specific criteria; for example those that match the NICE reference case.

We will compare and contrast our findings with previously published reviews of HSUVs for diabetic retinopathy. We will discuss the strengths and limitations and highlight any apparent gaps in the identified evidence. We will also identify the strengths and limitations of our review and make suggestions for future research.

This protocol is the first of its kind to be published, and the first to be registered prospectively. By creating a public record of the intended review process it is possible to maintain transparency in the process of selecting parameters to be used in decision analytic models of health technologies. We hope that this approach will become standard practice as part of the modeling process.

## Additional files

Additional file 1
**Appendix A: search terms.** This document sets out the search terms that we will use.

Additional file 2
**Appendix B: data extraction form.** This document provides a screenshot of the electronic data extraction form that we will use.

## Abbreviations

DR: Diabetic retinopathy
HSUV: Health state utility value
NDESP: NHS Diabetic Eye Screening Programme
NHS: National Health Service (England)
NICE: National Institute for Health and Care Excellence
NPDR: Non-proliferative diabetic retinopathy
PDR: Proliferative diabetic retinopathy
PRISMA: Preferred reporting items for systematic reviews and meta-analyses
QALY: Quality-adjusted life year
