# Supplementary material for: Health state utility values for diabetic retinopathy: protocol for a systematic review and meta-analysis
Source: Syst Rev. 2015 Feb 21;4:15. doi: 10.1186/s13643-015-0006-6 (PMC4342097; doi:10.1186/s13643-015-0006-6)
Supplement: Additional file 1 — Appendix A: search terms. This document sets out the search terms that we will use. [file 13643_2015_6_MOESM1_ESM.pdf]

Appendix A: search terms

1. exp diabetic retinopathy/
2. (diabetic retinopathy or stdr or dmo or dme).af.
3. diabet$.af.
4. (maculopathy or macular edema or macular oedema or csmo or csme).af.
5. 3 and 4
6. 1 or 2 or 5
7. exp quality adjusted life year/
8. quality adjusted life$.af.
9. disability adjusted life$.af.
10. (daly$ or qaly$ or qald$ or qale$ or qtime$).af.
11. hsuv.af.
12. health status/
13. health state.af.
14. (sf6d or sf 6d or short form 6d or shortform 6d or sf six d or sfsixd or shortform six d or short form six d).af.
15. (euroqol or euro qol or euro-qol or eq5d$ or eq 5d$ or eq-5d$ or rosser).af.
16. (hql or hqol or hrqol or hrql).af.
17. (healthy years equivalent or hye$).af.
18. (hui or hui1 or hui2 or hui3).af.
19. (15d or 15 d).af.
20. aqol$.af.
21. addqol$.af.
22. disutilit$.af.
23. (qwb or wellbeing or well-being or well being).af.
24. (standard gamble or sg).af.

25. (time trade off or time trade-off or time tradeoff or tto).af.
26. (person trade off or person trade-off or person tradeoff or pto).af.
27. (visual analogue scale or vas).af.
28. exp quality of life/
29. (quality adj2 life).af.
30. (cost utility or cost-utility or cua).af.
31. 7 or 8 or 9 or 10 or 11 or 12 or 13 or 14 or 15 or 16 or 17 or 18 or 19 or 20 or 21 or 22 or 23 or 24 or 25 or 26 or 27 or 28 or 29 or 30
32. 6 and 31
