# Supplementary material for: Health state utility values for diabetic retinopathy: protocol for a systematic review and meta-analysis
Source: Syst Rev. 2015 Feb 21;4:15. doi: 10.1186/s13643-015-0006-6 (PMC4342097; doi:10.1186/s13643-015-0006-6)
Supplement: Additional file 2 — Appendix B: data extraction form. This document provides a screenshot of the electronic data extraction form that we will use. [file 13643_2015_6_MOESM2_ESM.pdf]

Study characteristics

Fill in this part of the form once for each study

*Required

1. **First author** *

2. **Publication year** *

3. **Article title** *

4. **Publication name** *
   e.g. "Diabetologia"

5. **Study design**
   Mark only one oval.
   - ( ) Clinical decision analysis (e.g. RCT)
   - ( ) Outcomes study
   - ( ) Other:

6. **Interventions / comparators**
   if appropriate

7. **Study sample size**
   Total across all group

8. **Inclusion / exclusion criteria**

9. **Response rates**

10. **Loss to follow-up**

11. **Missing data**
What percentage of data were missing and how did the author(s) address this?

12. **Cooper rank**
Refer to Cooper et al (2005)
Mark only one oval.

- 1
- 2
- 3
- 4
- 5
- 6

13. **Total number of HSUVs reported in paper**
*

HSUV specifics

Fill in this part of the form once for each separately reported HSUV

14. **Reported HSUV point estimate type**
Mark only one oval.

- ( ) Mean
- ( ) Median

15. **Reported HSUV** *

....

16. **Reported measure of variance type**
Mark only one oval.

- ( ) Standard deviation
- ( ) Variance
- ( ) Other: ....

17. **Reported variance statistic**

....

18. **Retinopathy state** *
as described in the study

....

19. **Maculopathy state**
as described in the study

....

20. **Grading system**
The retinopathy grading system to which this HSUV relates
Mark only one oval.

- ( ) NHS Diabetic Eye Screening Programme (NDESP)
- ( ) American Academy of Ophthalmology (AAO)
- ( ) Early Treatment Diabetic Retinopathy Study (ETDRS)
- ( ) Liverpool Diabetic Eye Study (LDES)
- ( ) Scottish Diabetic Retinopathy Grading Scheme (SDRGS)
- ( ) Royal College of Ophthalmologists (RCO)
- ( ) Other: ....

21. **Visual function measurement method**
Mark only one oval.

- ( ) LogMAR
- ( ) Snellen
- ( ) Other: ....

22. **Visual acuity/function level**

....

23. **Sample size**
For reported HSUV

....

24. **Sample country**

....

25. **Sample age range**

....

26. **Other sample specifics**

....
....
....
....
....

27. **Valuation method** *
Direct or indirect methods
Mark only one oval.

- ( ) Standard gamble
- ( ) Time trade-off
- ( ) Person trade-off
- ( ) Discrete choice experiment
- ( ) EQ-5D
- ( ) HUI3
- ( ) SF-6D
- ( ) 15D
- ( ) Mapping algorithm
- ( ) Visual analogue scale
- ( ) Other: ....

28. **Valuation source**
Mark only one oval.

- ( ) Patients
- ( ) Public
- ( ) Other: ....

29. **Value set country**

....

30. **Upper anchor**
Mark only one oval.

- ( ) "full health"
- ( ) Other: ....

31. **Lower anchor**
Mark only one oval.

- ( ) "worst health state imaginable"
- ( ) "dead"
- ( ) Other: ....

32. **Administration method**
Select all that apply
Tick all that apply.

- [ ] Face-to-face interview
- [ ] Telephone
- [ ] Web
- [ ] Self-complete
- [ ] Proxy
- [ ] Other: ....

33. **Study arm**
if applicable
Mark only one oval.

- ( ) Treatment
- ( ) Control
- ( ) Other: ....

34. **Treatment status**
if applicable
Mark only one oval.

- ( ) Pre-treatment
- ( ) Post-treatment
- ( ) Other: ....

35. **Mapped R0M0 value**

Mark only one oval.

- R0M0
- R0M1
- R1M0
- R1M1
- R2M0
- R2M1
- R3M0
- R3M1

Powered by
Google Forms
